# Semen and male genital tract characteristics of patients with Fabry disease: the FERTIFABRY multicentre observational study

**DOI:** 10.1186/s12610-019-0088-4

**Published:** 2019-05-15

**Authors:** Aline Papaxanthos-Roche, Aline Maillard, Lucie Chansel-Debordeaux, Martine Albert, Catherine Patrat, Olivier Lidove, Dominique P. Germain, Paul Perez, Didier Lacombe

**Affiliations:** 1 Reproductive biology laboratory-CECOS, Centre Aliénor d’Aquitaine, Hôpital Pellegrin CHU de Bordeaux, F-33000 Bordeaux, France; 2 Methodology and data management centre, USMR Unit, CHU de Bordeaux, F-33000 Bordeaux, France; 3 Reproductive biology unit, Centre Hospitalier Intercommunal Poissy/St-Germain-en-Laye, F-78300 Poissy, France; 4 UFR Université de Versailles St Quentin-en-Yvelines, F-78000 Versailles, France; 5 Histology, embriology, reproductive biology unit, Assistance Publique-Hôpitaux de Paris, Hôpital Cochin, F-75014 Paris, France; 6 Internal medicine department, Hôpital La Croix St-Simon, F-75020 Paris, France; 7 Sorbonne Universités UPMC, F-75005 Paris, France; 8 INSERM CNRS, Centre de recherche en Biologie, GH Pitié Salpêtriere, F-75013 Paris, France; 9 French Reference Centre for Fabry disease, Division of Medical Genetics Unit, CHU Raymond Poincaré, F-92380 Garches, France; 10 Inserm U1179, F-78180 Montigny-le-Bretonneux, France; 11 Medical Genetics Departement, CHU de Bordeaux, INSERM U1211, F-33076 Bordeaux Cedex, France

**Keywords:** Fabry disease, Abnormal category in the semen analysis, Infertility, Maladie de Fabry, caractéristiques séminales, infertilité

## Abstract

**Background:**

Fabry disease (FD) is a rare disorder caused by the deficient activity of α-galactosidase A (α-Gal A). This enzymatic deficit results in the cellular accumulation of globotriaosylceramide (GL-3 or Gb_3_) and related glycosphingolipids in practically all organs and tissues in the body. The identification of deposits of Gb_3_ at the reproductive tract level suggests that this part of the body might be involved. We undertook this study to assess the impact of Fabry disease in male gonadal function.

**Materials and methods:**

This was a multicentre cross-sectional, prospective study that included patients aged 18 to 65 years with Fabry disease, receiving care in a specialized institution. The prevalence of at least one abnormal category in the semen analysis was presented with 95% confidence intervals (CI). The association between infertility and semen analysis abnormality was assessed by Fisher’s exact test. The association of factors associated with fertility or semen analysis abnormality were analysed by a multivariable logistic regression model and expressed by an odds ratio (OR) and its bilateral 95% CI.

**Results:**

Overall, 14 (82.4% [95% CI, 56.6–96.2]) of the patients had at least one abnormal category in the semen analysis based on WHO criteria. Sixteen patients responded to the questionnaire on fertility, 11 of whom were classified as fertile. Nine of the 11 fertile patients presented at least one abnormal category in the semen analysis. No association was found between infertility and semen analysis abnormality (*p* = 1.0000). Age of patient at inclusion (OR, 1.19; 95% CI, 0.98 to 1.45; *p* = 0.0854) and duration of replacement therapy (OR, 1.28; 95% CI, 0.96 to 1.65; *p* = 0.1263) were associated with sperm abnormalities. Eleven of the 16 patients had a normal hormonal profile. An ultrasound anomaly of the genital tract was observed in 12 patients.

**Conclusions:**

These results suggest that, while FD might have a detrimental effect on the semen characteristics, the reproductive function diminished only slightly. Further studies are warranted to assess the impact of the disease and of sperm abnormalities in the fertility of male patients with FD.

## Resume

**Contexte:**

La maladie de Fabry (FD) est. une maladie rare de transmission génétique liée au chromosome X due à un déficit en α-galactosidase A (α-GAL A) lysosomale. Ce déficit enzymatique entraîne l’accumulation de globotriaosylcéramide (GL-3 ou Gb3) dans pratiquement tous les types cellulaires de l’organisme, responsable d’une atteinte multisystémique. Le retentissement sur l’appareil génital étant peu documenté, cette étude a pour objectif d’évaluer l’impact de la maladie de Fabry sur la fonction gonadique masculine.

**Matériels et méthodes:**

Il s’agit d’une étude observationnelle, prospective, transversale, multicentrique incluant tous les patients suivis dans des centres spécialisés, âgés de 18 à 65 ans, atteints de maladie de Fabry. La prévalence d’au moins une catégorie anormale dans l’analyse du sperme a été présentée avec des intervalles de confiance (IC) de 95%. L’association entre l’infertilité et l’anomalie du sperme a été évaluée par le test exact de Fisher. Les facteurs associés à l’anomalie du sperme ont été analysés par un modèle de régression logistique multivariée et estimés par des rapports de cotes (Odds ratio [OR]) et leurs IC 95%.

**Résultats:**

Au total, 14 patients [82.4% (IC 95%, 56.6–96.2)] présentaient au moins une caractéristique spermatique anormale selon les critères OMS. Seize patients ont répondu au questionnaire sur la fertilité, dont 11 ont été classés comme fertiles. Neuf des 11 patients fertiles présentaient au moins une anomalie des caractéristiques spermatiques. Aucune association n’a été trouvée entre l’infertilité et une analyse anormale du sperme (*p* = 1.0000). L’âge du patient à l’inclusion (OR, 1.19; IC 95%, 0.98–1.45; *p* = 0.0854) et la durée du traitement substitutif (OR, 1.28; IC 95%, 0.96–1.65; *p* = 0.1263) étaient associés à une anomalie des caractéristiques spermatiques. Onze des 16 patients avaient un profil hormonal normal. Une anomalie échographique du tractus genital était observée dans 12 des patients.

**Conclusions:**

Ces résultats suggèrent que bien que des anomalies des caractéristiques séminales puissent être observées chez des patients atteints de maladie de Fabry, la fonction de reproduction est. très peu altérée.

## Background

Fabry disease (FD) is a rare (frequencies of up to 1/22570 classic and 1/1390 later-onset phenotypes in males) X-linked lysosomal storage disorder caused by the deficient activity of α-galactosidase A (α-gal A) [1]. This enzymatic deficit results in the cellular accumulation of globotriaosylceramide (GL-3 or Gb_3_) and related glycosphingolipids in practically all organs and tissues in the body. Males are more severely affected than females. Clinical onset of the disease typically occurs during childhood or adolescence with recurrent episodes of severe, debilitating neuropathic pain in the extremities (acroparesthesia). Patients have marked accumulation of Gb_3_ and related glycolipids in capillaries and small blood vessels, which cause the major symptoms in childhood or adolescence. These include the acroparesthesia, angiokeratomas, anhidrosis or hypohidrosis, gastrointestinal symptoms including abdominal pain and cramping, and a characteristic corneal dystrophy (cornea verticillata). With increasing age, the systemic Gb_3_ deposition leads to arrhythmias, left ventricular hypertrophy and hypertrophic cardiomyopathy in the heart. In the kidneys, it leads to microalbuminuria, proteinuria, progressive renal insufficiency and then to renal failure. In the brain, there is a risk of cerebrovascular disease including transient ischemic attacks and strokes. Specific treatment of the disease is based in enzyme replacement therapy (ERT) or migalastat therapy. While the former reduces the accumulation of the glycolipids in cells, including the cells of the kidney and other organs, the latter has been found to substantially increases alpha-galactosidase-A activity, stabilizes related serum biomarkers, and improves cardiac integrity [2].

The identification of deposits of Gb_3_ at the reproductive tract level by optical and electron microscopy suggests that this part of the body might be involved [3–5]. A few case reports have been published in the literature. Very recently, an analysis of self-reported reproductive data from a large population (376 patients) of males and females with FD study carried out by Laney et al. revealed an increased reproductive fitness in this population. Nevertheless, detailed assessments on the impact of FD on the morphology and function of the reproductive system are scarce [6].

Based on these data, and given the low prevalence of FD, we launched a national multicentre cross-sectional study in male FD patients. The main objective of the study was to estimate the prevalence of sperm abnormalities in patients with FD. Secondary goals included assessing the impact of these abnormalities on fertility, to study the influence of: age, severity of the disease, and treatment by enzyme replacement therapy on the presence of sperm anomalies and on fertility. An etiopathogenic diagnosis by the study of the pituitary gonadotroph function and the morphological study of the reproductive system by ultrasound scan were also carried out.

## Patients and methods

This French study of male patients with Fabry disease was carried out in three specialized centres (Garches reference centre, Bordeaux University Hospital and the Hôpital de la Croix Saint Simon) between February 2009 and October 2013. The study was approved by the Interregional ethics committee SOOM III (N° 2008- AO1307–48). The study was registered with the European Union Drug Regulating Authorities for Clinical Trials and assigned the EudraTC No 2008-A01307–48.

Before enrolment, all patients gave informed consent. Eligibility criteria included patients aged 18 to 65 years with Fabry disease, receiving care in a specialized institution. The disease was proven by the deficient activity (< 12%) of the enzyme alpha-galactosidase A (α-Gal A) in leukocytes. Patients under judicial protection or not affiliated to the French social security were not included in the study.

### Study design

This was a national cross-sectional multicentre study. It included consecutively male patients with Fabry disease, regardless of whether it was the initial diagnosis or a follow-up visit, and regardless of whether they were under treatment or not. Men donated semen samples after 3 to 5 days of sexual abstinence. Semen samples were analysed in three laboratories specialized in reproductive biology that were accredited according to the ISO 15189 standard for the performance of the analyses. All three laboratories undergo the same external quality control checks twice a year on semen analyses and sperm morphology assessments. For each sample, the following characteristics were recorded: volume (mL), pH, sperm count (million/mL) and total sperm number (millions/ejaculate), sperm vitality, leucospermia, motility (initial and at 2 h) [7]. The morphology of the spermatozoa was assessed by only one laboratory. Characteristics were classified according to the World Health Organization classification as normal if [8]: volume more than 1.5 mL, pH greater than 7.1, sperm count more than 15 million/mL ejaculated, total sperm number more than 39 million spermatozoa per ejaculate, progressive motility value over 32%, vital spermatozoa over 58% and amount of white blood cells less than 1 million cells/mL. Sperm morphology was assessed according to the David criteria with a cut-off value of for normality of 15%, and the Multiple Anomalies Index (MAI) (< 1.6) [9].

The etiopathogenesis of possible sperm abnormalities was determined by analysing the endocrine function and by means of a genito-urinary ultrasound scan. Blood samples to evaluate the hormonal profile were collected during the inclusion visit. If laboratory standards were not specified, data were classified as missing. The genito-urinary ultrasound scan was carried out during the inclusion visit and included the following examinations: right and left testicular volumes (calculated length x width x depth × 0.71 in mm), the presence of micro calcifications (yes/no) in right and left testicles, presence of nodules (yes/no) in the right and left testicles, absence (yes/no) of right and left epididymis, absence (yes/no) of seminal vesicles, varicocele (yes/no), and prostate (normal/abnormal). The fertility of the patients was assessed during a consultation with a medical doctor specialized in reproductive health by means of a questionnaire and defined as presented in the Appendix.

### Statistical analyses

Patients’ demographic characteristics (age, height, weight) clinical features (heart rate and blood pressure) and medical history were described by mean (SD) or frequency (%). Sperm characteristics and infertility were described comprehensively. The primary endpoint was the prevalence of sperm abnormalities in the included patients. The prevalence of at least one abnormal category in the semen analysis was determined by the ratio between the number of patients presenting at least one abnormality and the total number of included patients. The prevalence of each abnormality was also calculated, and determined by the ratio between the number of patients with that abnormality and the total number of patients.

95% CI were estimated by the exact binomial method. Secondary endpoints were (i) the association between infertility and semen analysis abnormality; (ii) the association between potentially relevant variables and semen analysis abnormality; (iii) association between variables and infertility.

Infertility was defined as shown in the Appendix. The association between infertility and semen analysis abnormality was assessed by Fisher’s exact test. Variables included in the univariable analysis were age of patient at inclusion (OR defined for an increase of one year), severity of Fabry disease (severe vs non severe), kidney failure (yes vs no), hypertrophic cardiomyopathy (yes vs no), duration of replacement therapy (increase of one month), professional or domestic exposure to products impacting on fertility (yes vs no), tobacco smoking (yes vs no), alcohol consumption (yes vs no), duration of FD from first symptoms (increase of one year), age of patient at the beginning of replacement therapy (increase of one year), active compound (α-galactosidase vs β-galactosidase). Due to the size of the sample, we adopted a strategy that included clinically relevant variables in the multivariable logistic regression model. The association of each variable with fertility or semen analysis abnormality was expressed by an OR and its bilateral 95% CI. For quantitative variables, the assumption of log-linearity of the association was systematically checked.

All analyses were carried out using a SAS version 9.3 software (SAS Institute, Cary, NC).

## Results

Thirty-seven male patients with Fabry disease diagnosis established by a deficient activity of lysosomal α-gal A (< 12%) and α-gal A gene mutation analysis were invited to participate in the study. Nineteen out of the 37 patients refused to participate, the reason being that the patient did not want to collect semen. In the end, 18 patients were included in the study. One patient left the study after signing the informed consent. Study duration was 59 months. The demographic and clinical characteristics of the patients are presented in Table 1. All patients included in the study presented mutations causing the classical phenotype.

**Table 1:** Patients’ characteristics and medical history

Demographics	n (md)	Mean (SD)
Age at inclusion (years)	17 (1)	41.5 (11.3)
Height (cm)	17 (1)	176.7 (6.5)
Weight (kg)	17 (1)	72.8 (13.1)
Heart rate (bpm)	15 (3)	67.1 (14.8)
Systolic blood pressure (mm Hg)	15 (3)	123.0 (15.9)
Diastolic blood pressure (mm Hg)	15 (3)	77.6 (13.7)
Medical history	n (md)	n (%)
Angiokeratomas	17 (1)	13 (76.5)
Kidney failure	17 (1)	7 (41.2)
Dialysis	7	4 (57.1)
Kidney transplantation	7	3 (42.9)
Coronary insufficiency	17 (1)	1 (5.9)
Hypertrophic cardiomyopathy	17 (1)	9 (52.9)
Replacement therapy	17 (1)	17 (100)
Duration of replacement therapy (years), mean (SD)	17 (1)	4.8 ± 4.6
Active compound	17 (1)	
α-galactosidase		10 (58.8)
β-galactosidase		7 (41.2)

*Abbreviations*: *SD* = Standard deviation; *md* = Missing data

Overall, 82.4% (95% confidence interval [CI], 56.6–96.2) of the patients had at least one abnormal category in the semen analysis based on WHO criteria and the David classification for morphological abnormality. A total of 58.8% (95% CI, 32.9–81.6) and 41.2% (95% CI, 18.4–67.1) of the patients presented with a decrease in the sperm number per mL and per ejaculate, respectively. Progressive motility was normal in 75% (95% CI, 47.6–92.7) of the cases. The percentage of abnormal sperm morphology according to the David classification was 53.3% (95% CI, 26.6–78.7) (Table 2).

**Table 2:** Seminal characteristics

Variable		Total	% (95% CI)
Volume (mL)	n (md)	17 (1)	
	Mean (SD)	4.3 (3.1)	
	Abnormal (< 1.5 mL)	4	23.5 (6.8–49.9)
	Normal (≥1.5 mL)	13	76.5 (50.1–93.2)
pH	n (md)	17 (1)	
	Mean (SD)	7.9 (0.2)	
	Abnormal	0	
	Normal (> 7.2)	17	100 (80.5–100.0)
Total sperm number (Millions/ejaculate)	n (md)	17 (1)	
	Mean (SD)	181.3 (265.1)	
	Abnormal (< 39 Millions/ejaculate)	7	41.2 (18.4–67.1)
	Normal (≥39 Millions/ejaculate)	10	58.8 (32.9–81.6)
Sperm count (Millions/mL)	n (md)	17 (1)	
	Mean (SD)	34.9 (53.9)	
	Abnormal (< 15 M/mL)	10	58.8 (32.9–81.6)
	Normal (≥ 15 M/mL)	7	41.2 (18.4–67.1)
Sperm vitality (%)	n (md)	17 (1)	
	Mean (SD)	58.8 (27.8)	
	Abnormal (< 58%)	4	23.5 (6.8–49.9)
	Normal (≥ 58%)	13	76.5 (50.1–93.2)
Presence of leucospermia (n)	n (md)	17 (1)	
	Yes	1	5.9 (0.1–28.7)
	No	16	94.1 (71.3–99.9)
Progressive sperm motility (n)			
Grade a + b	n (md)	16 (2)	
	Mean (SD)	41.5 (21.2)	
	Abnormal (< 32%)	4	25 (7.3–52.4)
	Normal (≥ 32%)	12	75 (47.6–92.7)
Grade a + b + c	n (md)	16 (2)	
	Mean (SD)	48.8 (22.2)	
	Abnormal (< 40%)	3	18.8 (4.0–45.6)
	Normal (≥ 40%)	13	81.3 (54.4–96.0)
Sperm motility at 2 h (n)			
Grade a + b	n (md)	4 (14)	
	Mean (SD)	42.5 (32.4)	
	Abnormal (< 32%)	1	25 (0.6–80.6)
	Normal (≥32%)	3	75 (19.4–99.4)
Grade a + b + c	n (md)	4 (14)	
	Mean (SD)	49.3 (33.8)	
	Abnormal (< 40%)	1	25 (0.6–80.6)
	Normal (≥40%)	3	75 (19.4–99.4)
Sperm morphology (n)	n (md)	15 (3)	
	Mean (SD)	12.1 (8.7)	
	Abnormal (< 15%)	8	53.3 (26.6–78.7)
	Normal (≥15%)	7	46.7 (21.3–73.4)
MAI (n)	n (md)	15 (3)	
	Mean (SD)	1.7 (0.3)	
	Abnormal (≥1.6)	10	66.7 (38.4–88.2)
	Normal (< 1.6)	5	33.3 (11.8–61.6)

*Abbreviations:* md = missing data; CI = confidence interval; SD = standard deviation; MAI = Multiple Anomalies Index

Eleven (68.8%) out of 16 patients (hormonal data was missing for one patient) had a normal hormonal profile, four (36.4%) of which had sperm abnormalities (Table 3). The number of semen analysis abnormality cases increased twice (4 out of 5 patients, 80%) among patients with an abnormal hormonal profile (5 out of 16 patients, 31.3%). Overall, ultrasound scans revealed abnormalities in the genital tract morphology in 76.5% of the patients. The mean (standard deviation [SD]) volumes of the right and left testes were 10.2 mL (3.0) and 9.5 mL (3.1), respectively. Micro-calcifications were identified throughout both testicles in 41.2% (7 out of 17) of the patients. Testicular lumps were detected in 17.6% (right testicle) and 5.9% (left testicle) of the patients. There were 41.2 and 58.8% of patients with right and left epididymal cysts, respectively. Abnormal seminal vesicles were detected in 20% of patients, all of whom presented semen abnormalities. Varicocele affected 5 patients (1 right varicocele, 3 left varicocele and 1 right and left varicocele) and four of them presented sperm abnormalities. Prostate was normal in 64.7% of the patients.

**Table 3:** Hormonal profile

Variable		Total	Normal semen analysis	Abnormal category in the semen analysis
Total testosterone (n, %)	n (md)	10 (7)	4 (4)	6 (3)
	normal	10	4	6
Sex Hormone-binding globulin S-HBG) (nmol/L)	n (md)	9 (8)	2 (6)	7 (2)
	Normal	5	1	4
	Abnormal	4	1	3
Follicle stimulating hormone (FSH) (mU/mL)	n (md)	13 (4)	6 (2)	7 (2)
	Normal	6	2	4
	Abnormal	7	4	3
Luteinizing hormone (LH) (mU/mL)	n (md)	13 (4)	6 (2)	7 (2)
	Normal	12	6	6
	Abnormal	1	0	1
Inhibin B (pg/mL)	n (md)	10 (7)	5 (3)	5 (4)
	Normal	8	5	3
	Abnormal	2	0	2
17β estradiol (pmol/L)	n (md)	14 (3)	6 (2)	8 (1)
	Normal	6	2	4
	Abnormal	8	4	4
Prolactine (PRL) (pmol/L)	n (md)	9 (8)	6 (2)	3 (6)
	Normal	6	4	2
	Abnormal	3	2	1
Overall result	n (md)	16 (1)	8	8 (1)
	Normal	11	7	4
	Abnormal	5	1	4

*Abbreviations*: *md* = missing data

Sixteen patients responded to the questionnaire on fertility, of whom 11 (68.8%) were classified as fertile. Nine (81.8%) out of the 11 fertile patients presented at least one abnormal category in the semen analysis.

The five patients that had scored as “undetermined” in the fertility questionnaire were considered as infertile for the statistical model. Fisher’s exact test revealed no association between infertility and semen analysis abnormality (*p* = 1.0000).

Age of the patient at inclusion, age of patient at the beginning of therapy, duration of replacement therapy and alcohol consumption were associated to the presence of sperm abnormalities in univariable analysis. Variables included in the multivariable analysis due to its clinical relevance were age of patient at inclusion, duration of replacement treatment and active compound. Older patients at inclusion (odds ratio [OR], 1.19; 95% CI, 0.98 to 1.45. *p* = 0.0854) and patients with a longer duration of replacement therapy (OR, 1.28; 95% CI, 0.96 to 1.65; *p* = 0.1263) were more likely to present abnormalities in the semen analysis (Table 4).

**Table 4:** Association of variables with abnormalities in semen analysis

Variable	n		Univariable analysis			Final multivariable model (*n* = 17)		
			OR	95% CI	*p*-value	OR	95% CI	*p*-value
Age of patient at inclusion	17	For the increase of one year	1.16	1.01–1.33	***0.0403***	1.19	0.98–1.45	0.0854
Severity of FD	17	1 - Severe vs **0 - Non severe**	1.17	0.12–10.99	0.8927			
Kidney failure	17	1 - Yes vs **0 - No**	1.33	0.19–9.31	0.7717			
Hypertrophic cardiomyopathy	17	1 - Yes vs **0 - No**	0.48	0.07–3.35	0.4592			
Duration of replacement treatment	17	For the increase of 1 year	1.26	0.96–1.65	*0.0965*	1.28	0.93–1.74	0.1263
Professional or domestic exposure	17	1 - Yes vs **0 - No**	0.87	0.05–16.74	0.9292			
Tobacco consumption	17	1 - Yes vs **0 - No**	0.48	0.06–3.99	0.4939			
Alcohol consumption	17	1 - Yes vs **0 - No**	0.30	0.04–2.2	*0.2363*			
Duration of FD from first symptoms	16	For the increase of 1 year	1.05	0.96–1.16	0.2866			
Age of patient at the beginning of replacement therapy	17	For the increase of 1 year	1.09	0.98–1.22	*0.1222*			
Active compound	17	1 - α-galactosidase vs **2 - ß-galactosidase**	2.00	0.28–14.2	0.4882	3.77	0.21–66.8	0.3662

*Abbreviations: CI* = confidence interval; *OR* = odds ratio; *FD* = Fabry disease

The references for OR are in bold

Alcohol consumption (*p* = 0.2399) and a more severe FD (*p* = 0.1959) were found to increase the risk of infertility in the univariable model, although not in a statistically significant way (Table 5). Since no patient had the infertile status, no further multivariable analysis was carried out.

**Table 5:** Association of variables with fertility

Variable	n		Univariable analysis		
			OR	95% CI (OR)	p-value
Age of patient at inclusion	16	For the increase of one year	1.01	0.92–1.11	0.7839
Severity of FD	16	1 - Severe vs **0 - Non-severe**	0.15	0.01–2.29	*0.1724*
Duration of replacement treatment	15	For the increase of 1 year	1.00	0.98–1.02	0.8776
Professional or domestic exposure	16	1 - Yes vs **0 - No**	2.50	0.12–50.44	0.5501
Tobacco consumption	16	1 - Yes vs **0 - No**	0.44	0.04–5.40	0.5189
Alcohol consumption	16	1 - Yes vs **0 - No**	0.21	0.02–2.52	*0.2173*

*Abbreviations*: *OR* = Odds ratio; *CI* = confidence interval; *FD* = Fabry disease

The references for OR are in bold

## Discussion

We and others have reported individual cases of Gb_3_ (or GL-3) storage in the reproductive system of patients with FD, with inconsistent results regarding fertility. However, the association between the storage of Gb_3_ or the impact of FD on reproductive function has not been established in large FD populations [3–5, 10–12].

Patients included in this study presented classical symptoms including hypertrophic cardiomyopathy (52.9%), kidney failure (41.2%) and coronary insufficiency (5.9%). The incidence of dermatological and ophthalmological manifestations was slightly lower than that described in the literature. Overall, 76.5 and 47.1% of patients presented with angiokeratomas and cornea verticillata, respectively [13].

The progressive accumulation of Gb_3_ in the testes and epididymis has been associated with semen abnormalities [3, 4]. In our study, 82.4% of patients showed abnormalities in the semen analysis based on WHO criteria. Considering the parameters that are clinically significant for reproductive health, it was concluded that 52.9% of patients had at least one abnormality in semen. Most common alterations included a reduced sperm count (58.8% of patients), followed by semen volume and sperm vitality (23.5% for both). Progressive and total motility were altered for 25 and 18.8% of the patients, respectively. These results together with previous results suggest that FD might have a detrimental effect in the gonadal function [14]. Multivariable analysis revealed that severe (vs non-severe) disease was not associated with semen abnormalities. Only age of the patient at inclusion (*p* = 0.0854) and duration of ERT treatment (*p* = 0.1263) were associated in univariable analysis, but did not reach statistical significance. We investigated the nature of treatment impact by including the type of treatment as a variable in the multivariable analysis. There was no significant difference between patients treated with α-galactosidase and those treated with β-galactosidase. To our knowledge, this is the first study prospectively evaluating semen characteristics in Fabry patients.

Relating FD to male reproductive function is complex since the ability of a couple to conceive depends on both male and female factors. Fertility incidence in this study (68.8%) was consistent with the recent report of Laney et al. in which 64% of males with FD reported not having issues with infertility, but lower than the reported general population rate of 88–97.5% [15]. These results are in line with our analyses that reveal that severity of FD is not significantly associated with fertility (*p* = 0.1724) in univariable analysis. However, this result should be interpreted with caution due to small sample.

Also, four patients in this study received dialysis and three of them underwent a transplantation. Two of them were able to conceive without difficulty at least 6 years before the dialysis, and had two and three children respectively. Only one of them presented a sperm motility alteration, 7 years after the onset of renal failure. The other two patients were single. For one of them, an azoospermia was diagnosed at the age of 49, 15 years after the renal failure onset and after a cardiac transplantation. The second one had an oligozoospermia diagnosed 3 years after the onset of renal failure at the age of 52. The impact of renal failure on these alterations cannot therefore be ruled out and must be taken into consideration in patients with FD [16].

According to data published in the literature, Gb_3_ depositions in the endocrine system do not play a major clinical role in this disease in terms of hormone levels [17]. Our results were consistent with these findings in that the hormonal function of most patients was normal. Hauser et al. studied the impact of FD on endocrine organs and on the fertility fitness. The profile of gonadotropins and sex hormones as well as fertility rates in patients with FD were comparable to those of the Austrian general population [17]. Similarly, Faggiano et al. compared the function and morphology of endocrine glands among 18 patients with Fabry disease and age-matched healthy controls. Even though several endocrine dysfunctions were detected in FD patients, neither basal and stimulated FSH and LH levels nor sexual hormone levels differed significantly from controls [18].

Concerning morphology of the genital tract, over 76% of patients presented anomalies. Most common anomalies were micro-calcifications in both testes and epididymal cysts, the latter often resulting in obstructive azoospermia [19].

Potential limitations of our study include the low statistical power due to the poor patient recruitment rate. Some variables were included in the final multivariable model due to their clinical rather than statistical significance. Inclusion was challenging, and led to reducing the initially planned two semen analyses to a single one. In addition, the study reports on data from three centres, resulting in a lack of representativeness. On the other hand, while recent reports have been published on larger cohorts, the present study is of prospective nature. In addition, it provides a detailed assessment of morphology and function and a description of variables related to fertility.

## Conclusions

In conclusion, these results suggest that, while FD might have a detrimental effect on the semen characteristics of patients, the reproductive function remains only slightly diminished. Further studies are warranted to assess the extent of the disease in the fertility of male patients with FD.

## Appendix

**Table 6:** Fertility questionnaire

Infertility of current couple	Infertility of previous couple	Infertility of current partner	Infertility
Yes	Yes	No	Yes
Yes	No	No	Undetermined
Yes	No	Yes	Undetermined
Yes	Yes	Yes	Undetermined
No	Yes or no	Non-explored	No

## Abbreviations

CI: confidence interval
ERT: enzyme replacement therapy
FD: Fabry disease
GL-3 or Gb_3_: globotriaosylceramide
MAI: Multiple Anomalies Index
MD: missing data
OR: odds ratio
SD: standard deviation
α-gal A: α-galactosidase A
